# Nitrosative damage during retrovirus infection-induced neuropathic pain

**DOI:** 10.1186/s12974-018-1107-7

**Published:** 2018-03-05

**Authors:** Priyanka Chauhan, Wen S. Sheng, Shuxian Hu, Sujata Prasad, James R. Lokensgard

**Affiliations:** Department of Medicine, Neurovirology Laboratory, University of Minnesota Medical School, 3-107 Microbiology Research Facility, 689 23rd Ave. S.E, Minneapolis, MN 55455 USA

**Keywords:** LP-BM5, MAIDS, Neuropathic pain, Reactive gliosis, Nitrosylation, PD-1 KO

## Abstract

**Background:**

Peripheral neuropathy is currently the most common neurological complication in HIV-infected individuals, occurring in 35–50% of patients undergoing combination anti-retroviral therapy. Data have shown that distal symmetric polyneuropathy develops in mice by 6 weeks following infection with the LP-BM5 retrovirus mixture. Previous work from our laboratory has demonstrated that glial cells modulate antiviral T-cell effector responses through the programmed death (PD)-1: PD-L1 pathway, thereby limiting the deleterious consequences of unrestrained neuroinflammation.

**Methods:**

Using the MouseMet electronic von Frey system, we assessed hind-paw mechanical hypersensitivity in LP-BM5-infected wild-type (WT) and PD-1 KO animals. Using multi-color flow cytometry, we quantitatively assessed cellular infiltration and microglial activation. Using real-time RT-PCR, we assessed viral load, expression of IFN-γ, iNOS, and MHC class II. Using western blotting, we measured protein nitrosylation within the lumbar spinal cord (LSC) and dorsal root ganglion (DRG). Histochemical staining was performed to analyze the presence of CD3, ionized calcium binding adaptor molecule (Iba)-1, MHCII, nitrotyrosine, isolectin B4 (IB4) binding, and neurofilament 200 (NF200). Statistical analyses were carried out using graphpad prism.

**Results:**

Hind-paw mechanical hypersensitivity observed in LP-BM5-infected animals was associated with significantly increased lymphocyte infiltration into the spinal cord and DRG. We also observed elevated expression of IFN-γ (in LSC and DRG) and MHC II (on resident microglia in LSC). We detected elevated levels of 3-nitrotyrosine within the LSC and DRG of LP-BM5-infected animals, an indicator of nitric oxide (NO)-induced protein damage. Moreover, we observed 3-nitrotyrosine in both small (IB4^+^) and large (NF200^+^) DRG sensory neurons. Additionally, infected PD-1 KO animals displayed significantly greater mechanical hypersensitivity than WT or uninfected mice at 4 weeks post-infection (p.i.). Accelerated onset of hind-paw hypersensitivity in PD-1 KO animals was associated with significantly increased infiltration of CD4^+^ and CD8^+^ T lymphocytes, macrophages, and microglial activation at early time points. Importantly, we also observed elevated levels of 3-nitrotyrosine and iNOS in infected PD-1 KO animals when compared with WT animals.

**Conclusions:**

Results reported here connect peripheral immune cell infiltration and reactive gliosis with nitrosative damage. These data may help elucidate how retroviral infection-induced neuroinflammatory networks contribute to nerve damage and neuropathic pain.

## Background

The life-saving effects of combination anti-retroviral (cART) therapy against HIV-1 have led to greater appreciation of the deleterious, long-term consequences of chronic viral infection-related complications; including neurological disorders, opportunistic infections, and tumors. One major consequence is peripheral neuropathic pain, which is currently the most common neurological complication in HIV-infected individuals occurring in an estimated 35–50% of patients undergoing cART [1–4]. Patients with distal symmetrical polyneuropathy (DSP), the most common peripheral neuropathy occurring with HIV infection, report debilitating effects on their quality of life such as painful, abnormal touch sensations (dysesthesias), burning, pins-and-needles sensations, and numbness which are often associated with mechanical allodynia [5]. These symptoms generally begin distally on the soles of the feet and are symmetrically distributed to legs, with upper extremities being affected later [6]. It is well-known that several ART drugs themselves can contribute to DSP [7–10]. But, in addition to neurotoxic drugs, antiviral responses and their associated inflammation are also known to induce neurotoxic mediators which have been linked to DSP [11]. Therefore, the urgent need to understand HIV-DSP pathogenesis, identify risk factors in addition to neurotoxic drugs, and develop effective preventative strategies will intensify as cART patients live longer.

Despite its clinical significance, neuropathic pain in the context of viral infection is still an under-studied area, in part because appropriate experimental animal models have not yet been developed. A number of murine, feline, and non-human primate models have been developed for investigation of HIV DSP [12–17]. However, most of these models are expensive and the wide array of powerful, transgenic, and knockout animals used to assess mechanisms in vivo are not available. In this study, we have employed the model of murine-acquired immunodeficiency syndrome (MAIDS), where mice are infected with the LP-BM5 retroviral mixture. LP-BM5 infection is a chronic persistent infection and mice develop an immunodeficiency syndrome, hence termed MAIDS. Mice never recover from infection and eventually succumb to infection by 10–14 weeks. Most importantly, it has been reported that infected animals display symptoms of peripheral neuropathy by 6 weeks post-infection [18, 19]. LP-BM5 rapidly infiltrates the CNS generating encephalitis, blood brain barrier dysfunction, and spacial memory deficits within 6–8 weeks post-infection [18, 20, 21]. The LP-BM5 retroviral mixture includes a non-pathogenic ecotropic (BM5eco) helper virus and the pathogenic yet replication-defective (BM5def) virus. While BM5def induces disease, it requires BM5eco to replicate [22]. While not a perfectly accurate representation of human AIDS, LP-BM5 replicates in murine cells, induces lymphopenia, immunodeficiency, and chronic immune dysregulation, including polyclonal B-cell activation, hypergammaglobulinemia, enhanced susceptibility to opportunistic pathogens, and the development of terminal B-cell lymphomas, all of which may contribute to DSP development [23]. Indeed, this infection model allows for the investigation of the immune mechanisms that drive chronic retroviral infection-induced neuropathic pain [24]. Finally, this natural retroviral infection model allows the application of powerful knockout and transgenic murine tools to investigate infection-induced DSP.

Our laboratory has been investigating the role of activated, brain-infiltrating peripheral immune cells in driving chronic activation of brain-resident microglia following viral infection, particularly through production of IFN-γ [25–28]. Recent studies demonstrate that this type of chronic activation of resident glia is emerging as a common mechanism underlying various types of chronic pain [29, 30]. Similar to the brain, it is likely that dysregulated peripheral immune activation also promotes analogous activation of resident glia within the spinal cord, as well as DRG, leading to nerve damage, neurotoxicity, and neuropathic pain [31]. Moreover, the presence of nitrotyrosine, a marker of peroxynitrite formation, in nerve biopsies from patients with inflammatory neuropathies has been demonstrated [32]. Despite increased understanding of the mechanisms that drive neuropathic pain; the interplay between the immune and nervous systems remains unclear.

In this study, we evaluated the extent of chronic immune activation within the LSC and DRG in MAIDS animals with peripheral neuropathy and its associated nitrosative damage. Further, we assessed the role of the PD-1: PD-L1 negative immune checkpoint pathway in development of chronic neuropathic pain. The inhibitory co-receptor PD-1 plays an important role in regulating functional exhaustion of virus-specific T-cells during chronic infections [33–36]. Functional impairment of T-cells is characteristic of many chronic murine and human viral infections, including HIV/AIDS, due to the engagement of normal immune down-regulatory mechanisms, such as the PD-1/PD-L pathway [37]. A previous study from our laboratory has demonstrated the increased expression of PD-1 in MAIDS animals [26]. However, nothing is currently known regarding the development of neuropathic pain in PD-1 KO animals infected with LP-BM5. Here, we evaluated the development of neuropathic pain in PD-1 KO animals chronically infected with LP-BM5 and investigated its associated immune mechanisms and protein nitrosylation.

## Methods

### Ethical approval

This study was carried out in strict accordance with recommendations in the Guide for the Care and Use of Laboratory Animals of the National Institutes of Health. The protocol was approved by the Institutional Animal Care and Use Committee (protocol number: 1709-35110A and breeding protocol number: 1702-34587A) of the University of Minnesota. All animals were routinely cared for according to the guidelines of Research Animal Resources (RAR), University of Minnesota. Animals were sacrificed after isoflurane inhalation, whenever required and all efforts were made to ameliorate animal suffering.

### Virus and animals

The LP-BM5 retrovirus mixture was obtained from the NIH AIDS reagent program (Germantown, MD, USA). LP-BM5 viral stocks were prepared as described previously [26]. Virus stocks used for infection were produced as cell-free supernatants of SC-1 cells. Titers were determined by a standard retroviral XC plaque assay for the BM5eco virus.

Pathogen free C57BL/6 (Jackson Laboratories, Wilmington, MA, USA) or PD-1 KO animals (kindly provided by Sing Sing Way, Cincinnati Children’s Hospital) were housed in individually ventilated cages and were provided with food and water ad libitum at the RAR facility, University of Minnesota. Female mice were inoculated via the intraperitoneal (i.p.) route with two doses (2 × 10^4^/PFU dose) of LP-BM5 retrovirus mixture in 500 μl, with 3 days between doses [26].

### Assessment of mechanical allodynia

Tests of hind-paw mechanical hypersensitivity were conducted every week after 4 weeks of LP-BM5 infection until the designated end-point. Prior to testing, mice were allowed 20 min to habituate in the testing apparatus. Both left and right hind-paws were tested. Mechanical allodynia was assessed by using MouseMet electronic Von Frey system (Topcat Metrology, Cambridge, UK). At least six bilateral measurements were taken for each animal. Data were calculated as the paw withdrawal threshold in grams of force. The behaviorist was blinded to the animal condition.

### Isolation of leukocytes from spinal cord and DRG for flow cytometry

Mononuclear cells were isolated from the spinal cord of LP-BM5-infected wild-type (WT) and PD-1 KO mice using a previously described procedure with minor modifications [38–40]. In brief, whole tissues were harvested, (*n* = 4–6 animals/group/experiment), were minced finely using a scalpel in RPMI 1640 (2 g/L D-glucose and 10 mM HEPES), and were digested in 0.0625% trypsin (in Ca/Mg-free HBSS) at room temperature for 20 min. Single cell preparations of infected tissues were suspended in 30% Percoll and were banded on a 70% Percoll cushion at 900×*g* for 10 min at 15 °C. Total leukocytes obtained from the 30–70% Percoll interface were collected and counted on a hemocytometer using trypan blue dye exclusion method. To isolate mononuclear cells from DRG, we employed a non-enzymatic dissociation protocol described previously [41]. Briefly, six ganglia (L3-L5) were collected in a solution containing 1× HBSS/25 mM HEPES/10% FBS/10 μg/ml DNase (*n* = 4–6 animals/group/experiment). Animals were perfused with 1× HBSS and anesthetized using isoflurane before dissecting out the DRGs. Dissection was followed by homogenization of tissues with 1 ml syringe attached with 21 G needle and then 23 G needle. The homogenized solution was filtered through a cell strainer, and the cells were counted as described previously.

Following preparation of single cell suspensions, cells were treated with Fc block (anti-CD32/CD16 in the form of 2.4G2 hybridoma culture supernatant with 2% normal rat and 2% normal mouse serum) to inhibit nonspecific Ab binding. Cells were then counted using the trypan blue dye exclusion method, and 1 × 10^6^ cells were subsequently stained with anti-mouse immune cell surface markers for 15 min at 4 °C (anti-CD45-PE-Cy5 (eBioscience, San Diego CA, USA), anti-CD11b-AF700 (eBioscience), anti-CD4-BV510 (BioLegend, San Diego CA, USA), anti-CD8-PE-Cy7 (eBioscience), and anti-MHC-II-PE (eBioscience). Control isotype Abs were used for all fluorochrome combinations to assess nonspecific Ab binding. 10^5^ cells were acquired per sample by using a BD FACS LSR flow-cytometer (BD Biosciences, San Jose CA, USA) by employing FACS DIVA software and were normalized to the total number of cells isolated from the spinal cord to calculate the number of CD4 T cells, CD8 T cells, and macrophages. Data were analyzed using FlowJo software (TreeStar, Ashland, OR, USA).

### Semi-quantitative real-time RT-PCR

Total RNA from LSC (L3-L5) or DRG (L3-L5) tissue was extracted using an RNeasy Lipid Tissue Mini Kit (Qiagen, Valencia, CA, USA). The cDNA was synthesized from total RNA (1 μg) using Superscript III reverse transcriptase (Invitrogen) and oligo d(T)_12–18_ primers (Sigma-Aldrich, St. Louis, MO, USA). PCR was performed with the SYBR Advantage qPCR master mix (ClonTech, Mountain View, CA, USA). The qPCR conditions were 1 denaturation cycle at 95 °C for 10 s; 40 amplification cycles of 95 °C for 10 s, 60 °C annealing for 10 s, and elongation at 72 °C for 10 s followed by 1 dissociation cycle (Stratagene, now Agilent Technologies, La Jolla, CA, USA). The relative expression levels were quantified using the 2^−∆∆Ct^ method [42] and were normalized to the housekeeping gene hypoxanthine phosphoribosyl transferase (HPRT). The primer sequences were 5′- TGCTCGAGATGTCATGAAGG -3′ sense, 5- AATCCAGCAGGTCAGCAAAG-3′ antisense for HPRT; 5′- ATGGCTGTTTCTGGCTGTTACTG-3′ sense, 5′-GACGCTTATGTTGTTGCTGATGG-3′ antisense for IFN-γ; 5′- CCAATGTGTCCATGTCATTT-3′ sense, 5′- CTTTCTCTCTCTGCTCATCGC -3′ antisense for BM5eco; 5′- GAGTGGCCAAGTTTCGATGTGG-3′ sense, 5′- CGGGGAAAAGGGAAGTGTCGAT-3′ antisense for BM5def; 5′-GACGCTCAACTTGTCCCAAAAC-3′ sense, 5′- GCAGCCGTGAACTTGTTGAAC-3′ antisense for MHCII and 5′- TGGCCACCTTGTTCAGCTACG-3′ sense, 5′- GCCAAGGCCAAACACAGCATA-3′ antisense for iNOS.

### Immunohistochemistry (IHC)

LSC (L3-L5) and DRG (L3-L5) tissues were harvested from both uninfected and LP-BM5-infected animals that were perfused with phosphate-buffered saline (PBS), 2% sodium nitrate, and 4% paraformaldehyde. Tissues were subsequently submerged in 4% paraformaldehyde for 24 h (LSC) or 2 h (DRG) and were transferred to 25% sucrose solution for 2 days prior to sectioning. After blocking (10% normal goat or donkey serum and 0.3% Triton X-100 in PBS) for 1 h at room temperature (RT), tissue sections were incubated overnight at 4 °C with either of the following antibodies: rat anti-mouse CD3 (10 μg/ml; R&D Systems Inc., Minneapolis, MN, USA), rat anti-mouse MHCII (10 μg/ml; eBioscience), rabbit antibody to Iba-1 (2 μg/mL; Wako Chemicals, Richmond, VA, USA), mouse antibody to nitrotyrosine (5 μg/ml; LifeSpan BioSciences,Inc., Seattle, WA, USA), mouse antibody to neuron specific class III βeta-tubulin (10 μg/ml; R&D Systems Inc.), and rabbit antibody to neurofilament 200 (NF200, 1:80, Sigma-Aldrich, Australia). For fluorescent detection, sections were incubated with Cy3-conjugated donkey anti-goat or donkey anti-rabbit Ab (Jackson Immunoresearch Labs), Alexa Fluor 488 conjugated donkey anti-mouse and/or Alexa Flour 546 conjugated donkey anti-rat antibodies (Molecular Probes).

For nitrotyrosine staining, tissue sections were pretreated using heat-induced epitope retrieval. After washing three times with TRIS-buffered saline (TBS), secondary Ab (goat anti-mouse IgG biotinylated; Vector Labs, Burlingame, CA, USA) was added for 1 h at RT followed by incubation with ABC (avidin-biotinylated enzyme complex, Vector Labs) solution. For isotype staining, mouse IgG was used as primary antibody. The peroxidase detection reaction was carried out using 3,3′-diaminobenziding tetrahydrochloride (DAB; Vector Labs) for several minutes at RT. For nitrotyrosine and NF 200 double immunolabeling, tissue sections were incubated with both the primary antibodies simultaneously, overnight at 4 °C. After washing four times in PBS-T (PBS with 0.5% Triton X-100), sections were incubated for 1 h with Alexa Fluor 488 and/or Alexa Fluor 546 secondary antibodies (1:500, Molecular Probes). For nitrotyrosine and IB4 double staining, sections were stained with nitrotyrosine as described previously. After incubation with Alexa Fluor 546 goat anti-mouse secondary antibody (1:500, Molecular Probes), sections were washed twice with PBS-T and twice with PBS followed by a 2 h incubation with FITC-conjugated IB4 (1:100, Sigma Aldrich, Australia). After immunostaining, sections were washed in PBS-T four times and were counterstained with 1 μg/ml 4,6-diamidino-2-phenylindole (DAPI, Sigma Aldrich). Fluorescence was detected using appropriate filter combinations for DAPI, FITC/Alexa Fluor 488, Alexa Fluor 546/Cy3.

To evaluate the percentage of nitrotyrosine- and IB4- or NF200- double-positive sensory neurons, serial sections of DRGs (L3-L5) from three to four animals were cut (7 μm) and stained. At least four sections per DRG per animal were imaged and counted at × 20 magnification. The percentage of double-labeled neurons was calculated by dividing the total number of double-labeled neurons by the number of single IB4- or NF200-labeled neurons × 100.

### Western blotting

Animal tissues were harvested in T-PER (Thermo Scientific, Rockford, IL, USA) containing protease inhibitor (Sigma-Aldrich), were homogenized with a polytron on ice, and were centrifuged at 12,000×*g* for 20 min at 4 °C. Supernatants were collected and protein concentrations were measured with the Bio-Rad Protein Assay reagent (Bio-Rad Laboratories, CA, USA). Protein samples (45 μg) were mixed with 2× sample buffer (Bio-Rad Laboratories), were heated at 100 °C for 5 min and then were electrophoresed onto 4–20% pre-cast gels (Bio-Rad Laboratories) followed by transblotting to nitrocellulose membranes (0.45 μm). Membranes were rinsed in TTBS (Tris-HCl with NaCl and Tween 20) and were incubated in 5% blocking buffer (blotto in TTBS, Santa Cruz) for 1 h at room temperature before being probed with primary antibody (mouse anti-nitrotyrosine, MAB5404, 1:1000 in 1% blotto; Chemicon, now Millipore) overnight at 4 °C. After washing 3× with TTBS, membranes were incubated in alkaline phosphatase (AP) conjugated-secondary antibody (1:5000 in 1% blotto, Promega) at room temperature for 1 h. Membrane blots were washed 3× with TTBS followed by 2× assay buffer (1×) and then were incubated in substrate solution (CDP-Star, Applied Biosystems, now Thermal Fisher) for 10 min. The signal intensity of the protein bands was measured by employing Image Studio Lite software (LI-COR, Lincoln, NE, USA).

### Statistical analysis

One-way analysis of variance (ANOVA) with Tukey’s multiple comparison test was employed for graphical analysis. One-way ANOVA post hoc followed by Fisher’s PLSD test was used for the analysis of behavioral testing. Differences were considered significant, when *p* < 0.05. For statistical analysis and generation of graphs, Prism 5 software (Version 5.01; GraphPad Software Inc., CA, USA) was used.

## Results

### Establishment of LP-BM5 infection-induced neuropathic pain and its associated chronic immune activation

**Fig. 1:**
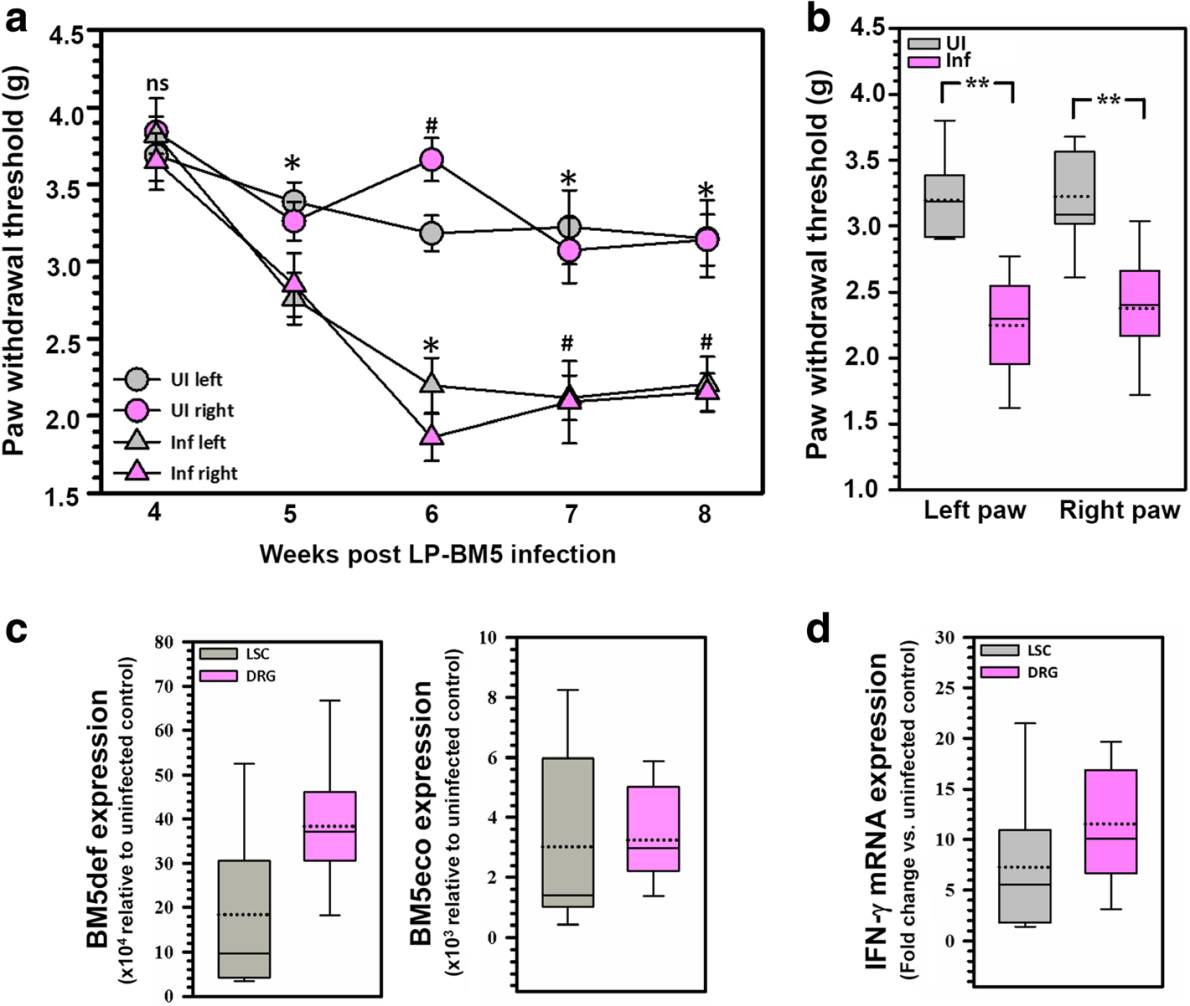
Establishment of LP-BM5 infection-induced neuropathic pain and associated chronic immune activation. **a** WT animals were randomly assigned to LP-BM5-infected (Inf) and uninfected (UI) groups (*n* = 10/group). Hind-paw withdrawal threshold (in grams) was assessed on both left and right paws at various time points via MouseMet electronic Von Frey. *p < 0.05 between infected (Inf) and uninfected (UI) WT animals, and #*p* < 0.05 between infected (Inf) and uninfected (UI) WT animals (left and right paws, respectively). Data are presented as mean ± SEM paw withdrawal threshold. **b** Hind-paw withdrawal threshold (in grams) was assessed on both left and right paws at 10 weeks p.i. via MouseMet electronic Von Frey (*n* = 10/group). **p < 0.01 between infected (Inf) and uninfected (UI) WT animals. Data are presented as mean ± SEM paw withdrawal threshold. **c** LP-BM5 viral loads were determined by measuring the levels of BM5def (disease-inducing virus) and BM5eco (helper virus) gag RNA in the LSC (L3 to L5) and DRG (*n* = 12) during MAIDS at 10 weeks p.i. via real-time RT-PCR. Results are presented as box plots of pooled data from individual animals. **d** Chronic IFN-γ mRNA production was measured in LSC (L3 to L5) and DRG (*n* = 12) at 10 weeks p.i. via real-time RT-PCR. Results are presented as box plots of pooled data from individual animals

Mice infected with the LP-BM5 retrovirus mixture have previously been reported to display symptoms of DSP by 6 weeks p.i. by Cao et al. [18]. We were able to repeat these findings using the MouseMet electronic von Frey system. LP-BM5-infected C57BL/6 mice exhibited hind-paw mechanical hypersensitivity after 5 weeks of infection, with no significant differences between the left and right hind-paws (Fig. 1a). Animals exhibited pain until 10 weeks post-infection when the majority of analyses were carried out (Fig. 1b). In addition, we also examined LP-BM5 retroviral load by measuring levels of BM5def (disease-inducing virus) and BM5eco (helper virus) gag RNA via real-time RT-PCR in the LSC and DRG of infected MAIDS animals and found high viral loads persisting within both tissues at 10 weeks p.i. (Fig. 1c). We also observed elevated mRNA levels of IFN-γ, 7-fold in LSC and 12-fold in DRG (Fig. 1d).

### Increased CD4^+^ and CD8^+^ T-cell infiltration into the spinal cord and DRG of MAIDS mice with DSP

**Fig. 2:**
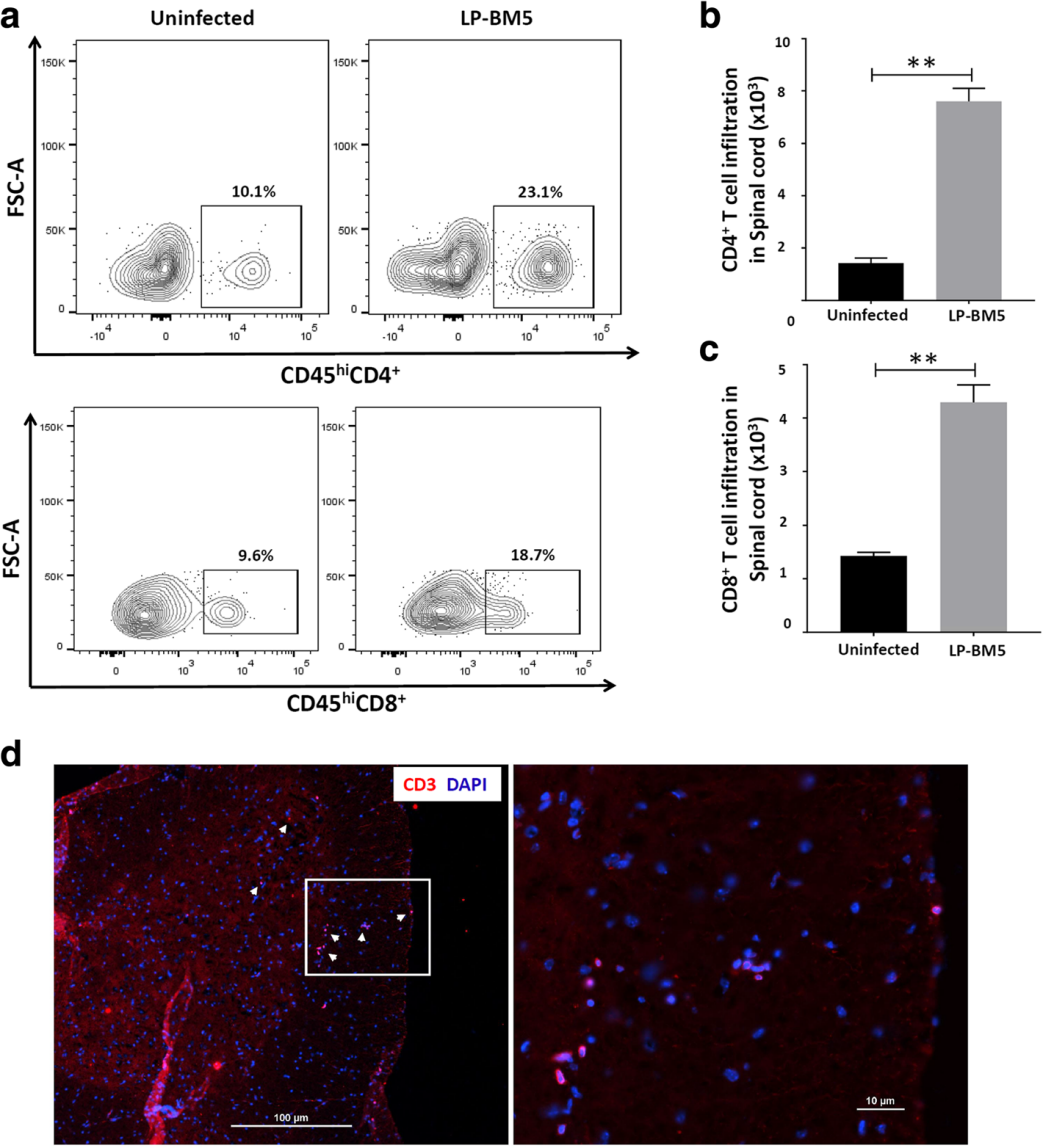
Increased infiltration of CD4^+^ and CD8^+^ T-cells into the spinal cord of LP-BM5-infected animals. Spinal cord-infiltrating leukocytes were banded on a 30–70% Percoll cushion, collected, and labeled with Abs specific for anti-CD45-PE-Cy5, anti-CD11b-AF700, anti-CD4-BV510, and anti-CD8-PE-Cy7 for analysis by flow cytometry. **a** Representative contour plots show the percentages of CD4^+^ and CD8^+^ T lymphocytes within uninfected and infected (LP-BM5) spinal cords of WT at 10 weeks p.i. **b** CD4^+^ T-cell infiltration into the spinal cord of uninfected and LP-BM5-infected animals. **c** CD8^+^ T-cell infiltration into the spinal cord of uninfected and infected animals. Pooled data present absolute numbers (mean ± SD) of CD4^+^ and CD8^+^ T-cells from two independent experiments using 4–6 animals per group. **p < 0.01. **d** IHC staining of infiltrating CD3+ T-cells in LSC (L3 to L5) sections from infected animals. White arrows indicate CD3^+^ T cells (red). Scale bar = 100 μm. The right panel indicates the × 40 image of the rectangular area. Scale bar = 10 μm

**Fig. 3:**
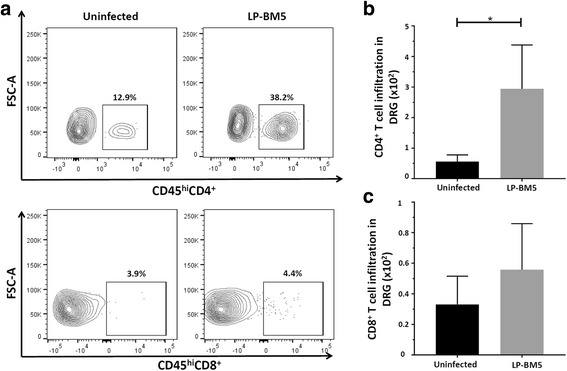
Increased infiltration of CD4^+^ T-cells into the DRG of LP-BM5-infected animals. Mononuclear cells from six ganglia (L3-L5) were isolated using a non-enzymatic dissociation method and were labeled with Abs specific for anti-CD45-PE-Cy5, anti-CD4-BV510, and anti-CD8-PE-Cy7 for analysis by flow cytometry. **a** Representative contour plots show the percentages of CD4^+^ and CD8^+^ T lymphocytes within uninfected and infected (LP-BM5) DRG of WT animals at 10 weeks p.i. **b** CD4^+^ T-cell infiltration into the DRG of uninfected and LP-BM5-infected animals. **c** CD8^+^ T-cell infiltration into the DRG of uninfected and infected animals. Pooled data present absolute numbers (mean ± SD) of CD4^+^ and CD8^+^ T-cells from two independent experiments using 4–6 animals per group. *p < 0.05

We next examined the infiltration of CD4^+^ and CD8^+^ T lymphocytes into the spinal cord of animals with DSP at 10 weeks p.i. Representative flow cytometry plots (Fig. 2a) show that LP-BM5 infection resulted in an increased frequency of both CD4^+^ and CD8^+^ T-cells within the spinal cord. As shown in Fig. 2b, c, mice infected with LP-BM5 exhibited significant increase in the absolute numbers of CD4^+^ and CD8^+^ T-cells in spinal cord when compared to uninfected mice. Furthermore, IHC staining of LSC sections from infected animals confirmed the presence of infiltrating CD3^+^ T-cells (white arrows) in the meningeal area, dorsal, and ventral horn of spinal cord (Fig. 2d). We did not observe any significant difference in the total number of myeloid cells in uninfected and LP-BM5-infected animals. In addition to spinal cord, we also carried out flow cytometric analyses of DRG tissues to assess infiltration of CD4^+^ and CD8^+^ T lymphocytes. In these studies, we observed a significant increase in the number of CD4^+^ T-cells infiltrating the DRG (Fig. 3a, b). However, we did not observe increased, infection-induced CD8^+^ T-cell infiltration (Fig. 3a, c).

### Activation of resident microglial cells in the spinal cords of MAIDS mice with DSP

**Fig. 4:**
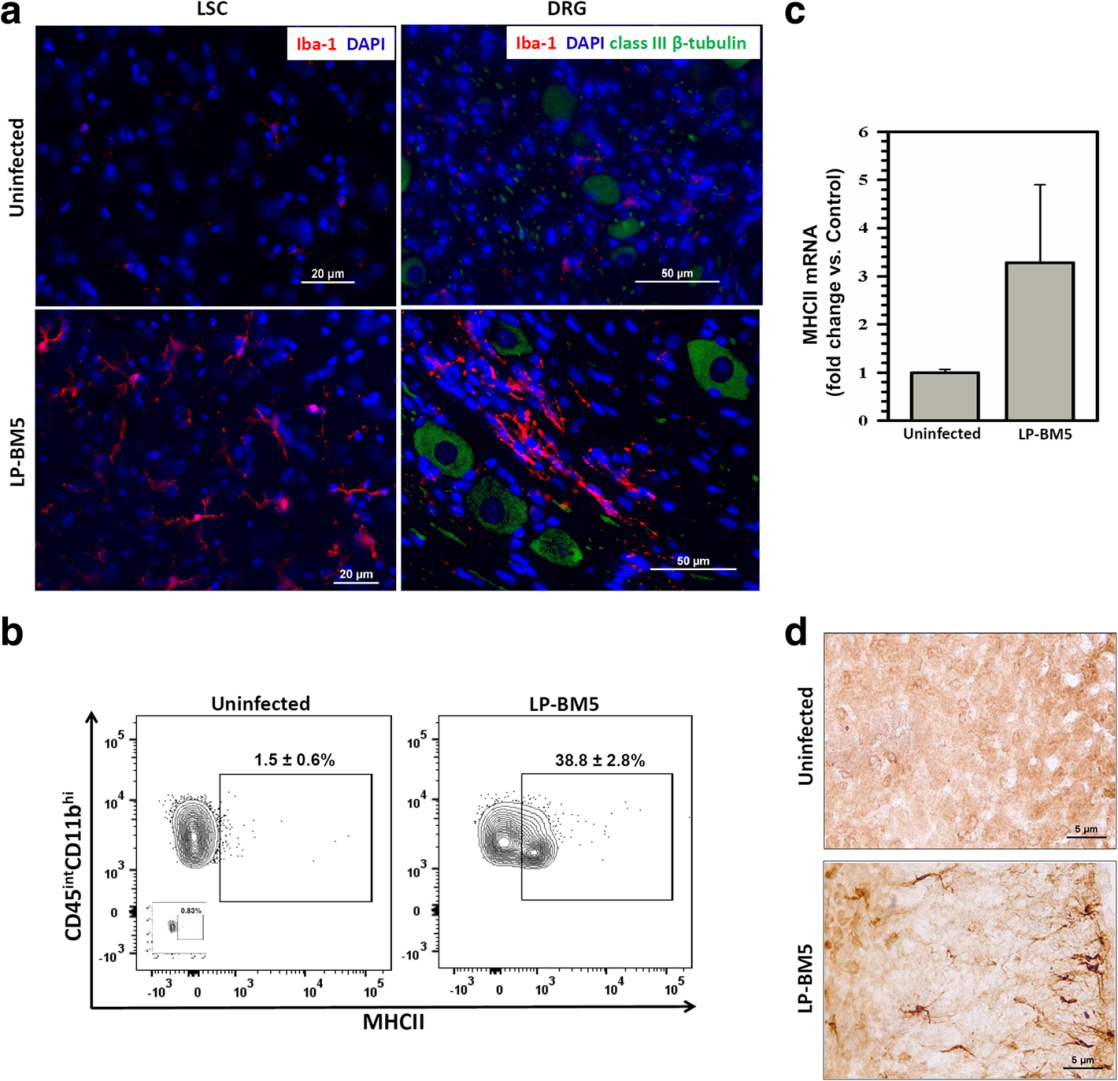
Chronic activation of resident microglial cells in LP-BM5-infected animals. **a** IHC staining of the macrophage/microglial cell marker, Iba-1 (red) within infected LSC (L3-L5), and DRG at 10 weeks p.i. Green staining is for neuron-specific class III β-tubulin. Blue is DAPI staining. Scale bar = 20 μm for LSC and 50 μm for DRG. **b** Resident microglial cells in the spinal cord of uninfected and infected animals were analyzed for expression of the activation marker MHCII. Contour plots showing the frequency of microglial cells expressing MHCII within uninfected and infected (LP-BM5) spinal cords of animals at 10 weeks p.i. Pooled data present the frequency (mean ± SD) of microglial cells expressing MHCII in spinal cord of uninfected and infected animals at 10 weeks p.i. from two independent experiments using 4–6 animals per group. Inset, isotype control. **c** MHCII expression was measured by real-time RT-PCR in the LSC of uninfected and infected (LP-BM5) animals (*n* = 4–6) at 10 weeks p.i. **d** IHC staining of MHCII (brown) in LSC sections from uninfected and infected (LP-BM5) animals. Scale bar = 5 μm

We next assessed activation of resident microglial cells in the LSC by determining levels of markers for reactive gliosis [25, 28, 43]. Firstly, we have used Iba I for IHC staining of infected LSC and DRG tissues, a marker that will stain both the microglia and infiltrating macrophages. IHC staining (red) confirmed the presence of both microglia and macrophages within infected LSC and DRG at 10 weeks p.i. Staining for neuron-specific class III β-tubulin was also performed (green), (Fig. 4a). This was followed by quantification of the CD45^int^CD11b^hi^ population of microglial cells and their expression of MHCII in the spinal cord of MAIDS mice using flow cytometry. We observed 38.8 ± 2.8% of microglial cells expressing MHCII with LP-BM5-infected mice as compared to 1.5 ± 0.6% microglial cells of uninfected mice (Fig. 4b). These data were substantiated using real-time RT-PCR for MHCII mRNA expression (Fig. 4c) and IHC staining for MHCII (brown) (Fig. 4d), both indicating the upregulation of MHCII in the LSC of mice infected with LP-BM5 at 10 weeks p.i. We did not observe a CD45^int^CD11b^hi^ population in DRG.

### Nitric oxide (NO)-mediated protein damage within the LSC and DRG of LP-BM5-infected animals

**Fig. 5:**
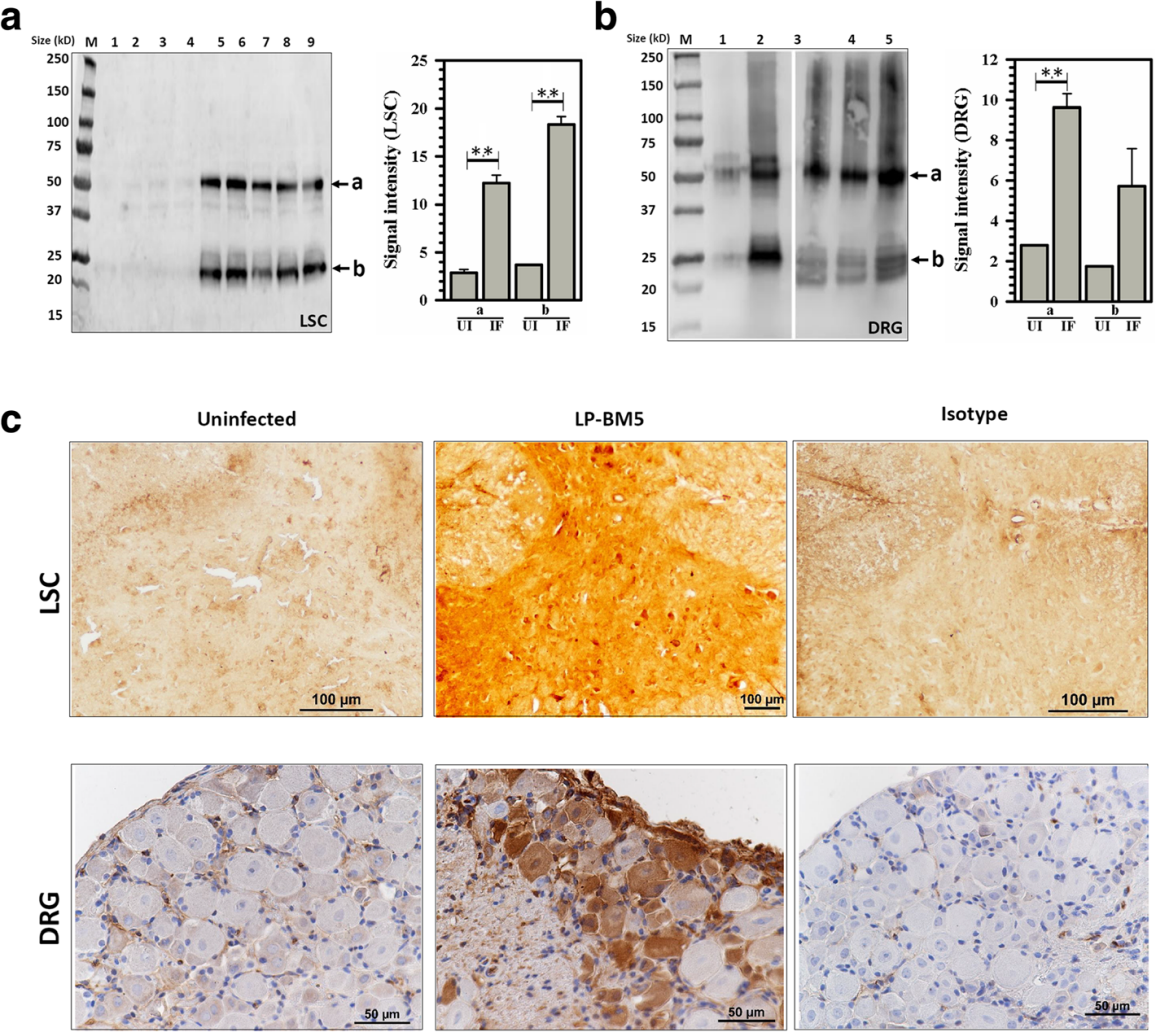
NO-induced damage within the LSC and DRG of LP-BM5-infected animals. **a** Western blot of LSC tissue lysates from uninfected (UI, lanes 1 to 4) and LP-BM5-infected (Inf, lanes 5 to 9) animals probed with anti-3-nitrotyrosine antibodies. Each lane displays tissue extract from one animal at 10 weeks p.i. The band labeled **a** identifies a high molecular weight (MW) protein band, while the **b** band points out a lower MW protein, both of which are nitrosylated. The signal intensity of both the **a** and **b** protein bands for uninfected (UI) and infected (IF) animals was measured using densitometry and plotted. **p < 0.01 (**b**) Western blot of lysates obtained from uninfected (UI, lane 1) and LP-BM5 infected (Inf, lanes 2 to 5) animals’ DRG (L3-L5) probed with anti-3-nitrotyrosine antibodies. Each lane displays a tissue extract pooled from three individual animals at 10 weeks p.i. The band labeled **a** represents a high MW protein, and the **b** band represents the lower MW protein band that are nitrosylated. The signal intensity of both **a** and **b** protein bands for uninfected (UI) and infected (IF) animals was measured and is plotted alongside. **p < 0.01 M = protein molecular weight marker ranging from size 15 to 250 kD. **c** IHC staining of LSC and DRG sections (L3-L5) from uninfected and infected (LP-BM5) mice with anti-3-nitrotyrosine antibody (brown) and mouse IgG isotype antibody. Scale bar = 100 μm for LSC and 50 μm for DRG

**Fig. 6:**
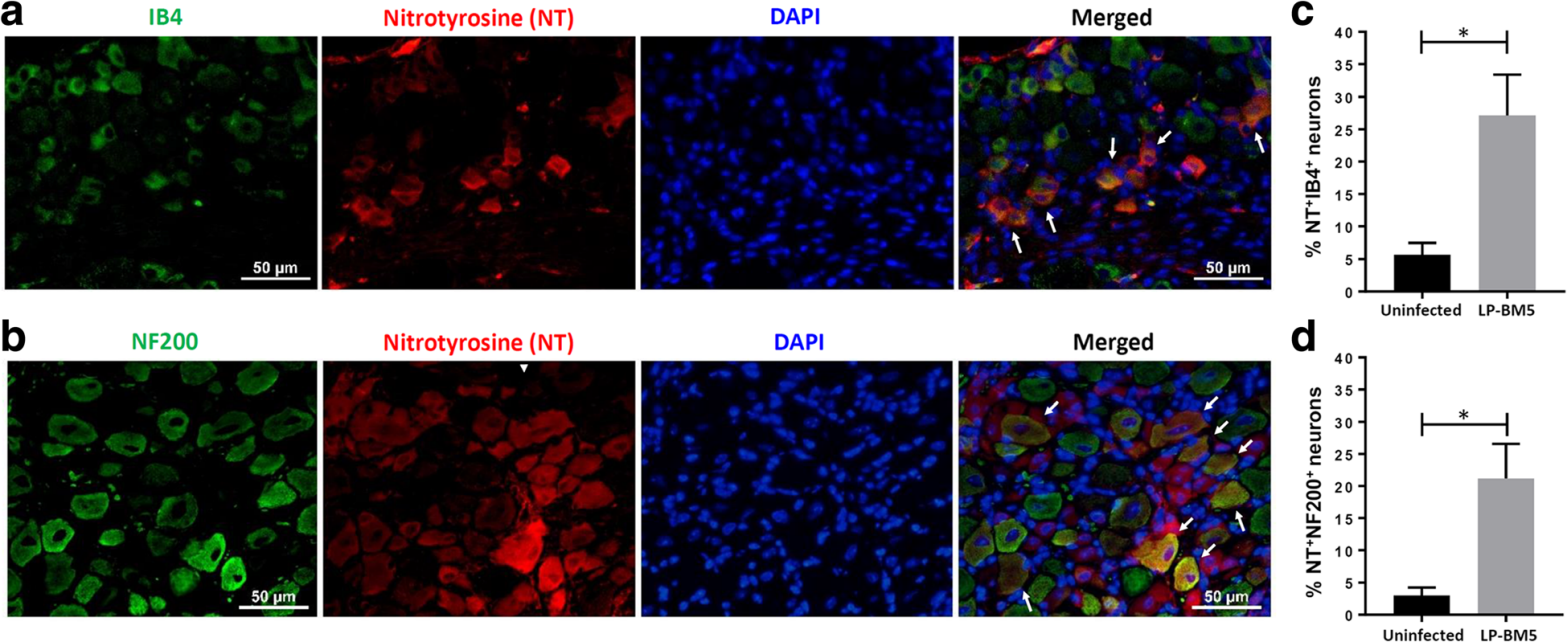
Nitrosative damage within DRG sensory neurons of LP-BM5-infected animals. Histochemistry to identify IB4-binding, NF200, nitrotyrosine, and DAPI was carried out on sections of DRG (L3-L5) at 10 weeks post-LP-BM5 infection. **a** Representative images depicting labelling of neuronal cells with IB4 (green), nitrotyrosine (red), DAPI (blue), and a merged image showing triple staining with IB4, nitrotyrosine, and DAPI. White arrows indicate the co-labeled neurons. **b** Representative images depicting labelling of neuronal cells with NF200 (green), nitrotyrosine (red), DAPI (blue), and a merged image showing triple staining with NF200, nitrotyrosine, and DAPI. White arrows indicate co-labeled neurons. Scale bar = 50 μm. **c** Graphical representation of the percentage of neurons showing double-immunolabeling for nitrotyrosine (NT) and IB4 in the DRG of LP-BM5-infected animals. **d** Column graph showing the percentage of neurons co-labeled with NT and NF200 in the DRG of LP-BM5-infected mice. *n* = 3–4 mice/group. Values are presented as mean ± SEM. *p < 0.05

Since neuropathic pain is a response to damage, we probed proteins in both LSC and DRG tissues from infected, as well as uninfected, animals for NO-mediated protein damage (i.e., protein nitrosylation) at 10 weeks post-LP-BM5 infection. We analyzed expression of 3-nitrotyrosine in MAIDS animals with DSP by both immunoblotting and IHC staining. We observed significantly greater infection-induced protein nitrosylation in the LSC (Fig. 5a), as well as DRG (Fig. 5b) from infected animals as compared to their uninfected littermates by immunoblotting. Interestingly, in both LSC and DRG, two major protein bands were nitrosylated following LP-BM5 infection. We quantified the signal intensity of both these bands (indicated a and b in Fig. 5a, b). Furthermore, LSC and DRG tissues from infected animals with MAIDS were cryosectioned and were stained with anti-nitrotyrosine Abs in situ (Fig. 5c). Positive staining cells (brown) were observed in LP-BM5-infected, but not uninfected sections of both LSC and DRG. Further characterization of the damaged neurons showed that 3-nitrotyrosine was co-localized in both IB4^+^, small and NF200^+^, large sensory neurons in the DRG of infected animals (Fig. 6a, b, respectively). We observed a significant increase in the percentage of IB4^+^ or NF200^+^ sensory neurons that were co-labeled with nitrotyrosine in LP-BM5-infected animals as compared to uninfected animals (Fig. 6c, d, respectively).

### Accelerated onset of hind-paw mechanical hypersensitivity in PD-1 KO animals infected with LP-BM5 and associated immune dysregulation

**Fig. 7:**
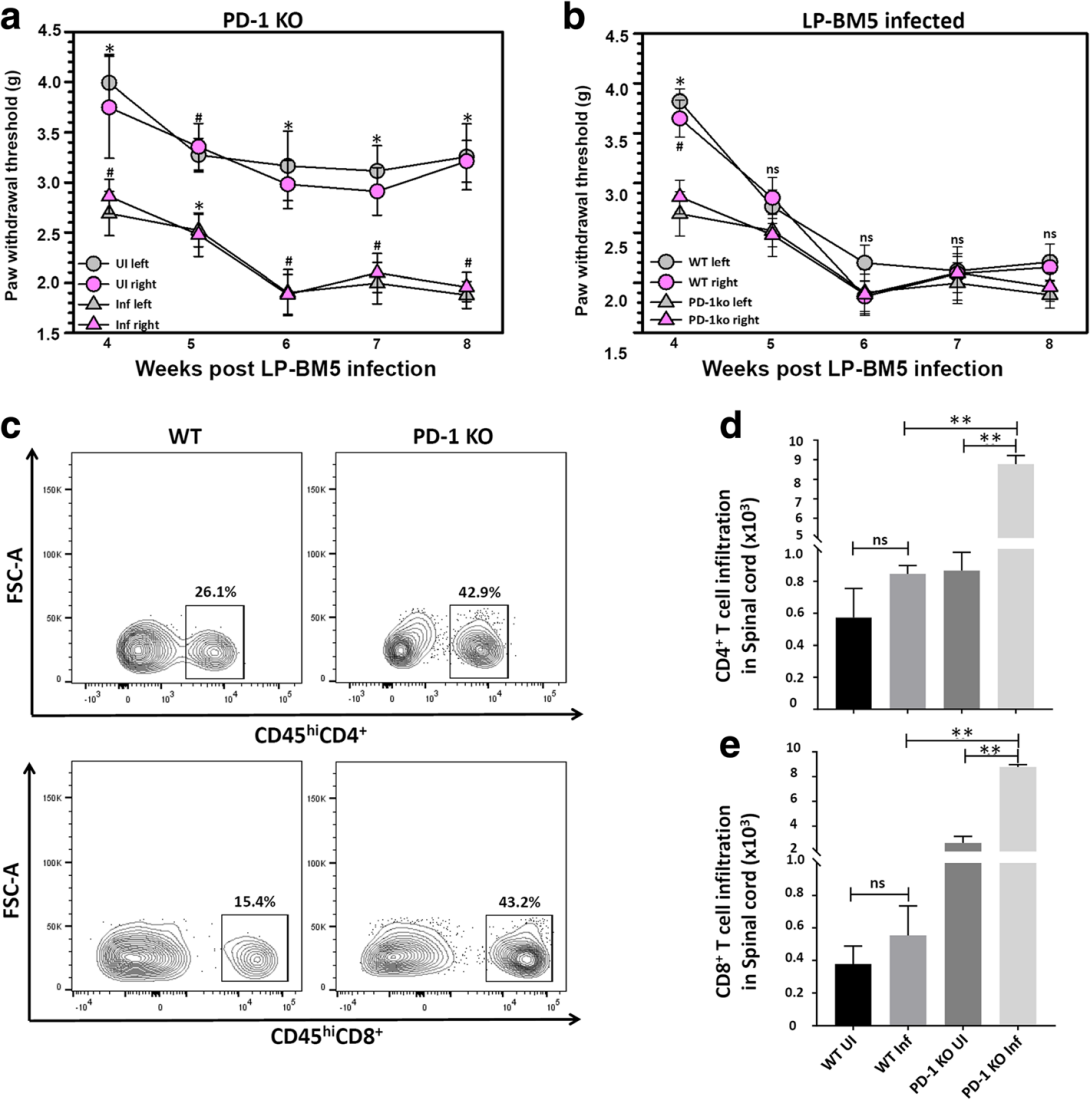
Accelerated onset of hind-paw mechanical hypersensitivity in PD-1 KO animals infected with LP-BM5 and associated cellular infiltration. Hind-paw withdrawal threshold was assessed in both WT, as well as PD-1 KO, animals as described in Fig. 1. **a** Paw withdrawal threshold (in grams) was assessed at various time points in LP-BM5-infected (Inf) PD-1 KO animals, as well their age-matched uninfected (UI) littermates (*n* = 10/group). *p < 0.05 between infected (Inf) and uninfected (UI) PD-1 KO animals, and #*p* < 0.05 between PD-1KO-infected (Inf) and uninfected (UI) PD-1 KO animals (left and right paws, respectively). **b** Paw withdrawal threshold was assessed at various time points in LP-BM5-infected WT, as well as PD-1 KO animals (*n* = 10/group). *p < 0.05 between infected PD-1 KO and infected WT animals, and #*p* < 0.05 between infected PD-1 KO and infected WT animals (left and right paws, respectively). Data are presented as mean ± SEM paw withdrawal threshold. **c** Representative contour plots show the percentages of CD4^+^ and CD8^+^ T lymphocytes within infected spinal cords of WT, as well as PD-1 KO animals at 4 weeks p.i. **d** CD4^+^ T-cell infiltration into the spinal cord of uninfected and infected WT and PD-1 KO animals. **e** CD8^+^ T-cell infiltration into the spinal cord of uninfected and infected WT and PD-1 KO animals. Pooled data present absolute numbers (mean ± SD) of CD4^+^ and CD8^+^ T-cells from two independent experiments using three animals per group. **p < 0.01

To determine if LP-BM5 would induce signs of peripheral neuropathy in chronically infected PD-1 KO animals, we examined hind-paw mechanical hypersensitivity as described in the previous section. No significant differences were found between the left and right hind-paws of infected or uninfected PD-1 KO animals. When infected PD-1 KO animals were monitored for mechanical allodynia, we observed a significant drop in paw withdrawal threshold at 4 weeks post-LP-BM5 infection and thereafter when compared with uninfected animals (Fig. 7a). Moreover, when compared with infected WT animals which developed mechanical allodynia at 5 weeks, PD-1 KO animals developed earlier mechanical allodynia by 4 weeks (Fig. 7b). No significant hind limb paralysis or weakness was observed in any of the infected mice. However, there was apparent splenomegaly and enlargement of lymph nodes in LP-BM5-infected animals.

We next examined the number of T lymphocytes infiltrating the spinal cord of WT and PD-1 KO mice to determine if it was associated with the earlier onset of mechanical hypersensitivity observed in PD-1 KO animals. Hence, we evaluated immune cell infiltrates in the spinal cord at 4 weeks p.i. The representative flow cytometry plots in Fig. 7c show that LP-BM5 infection resulted in an increased frequency of both CD4^+^ and CD8^+^ T-cells within the spinal cord of PD-1 KO animals as compared with WT mice. As shown in Fig. 7d, e, PD-1 KO mice infected with LP-BM5 exhibited a significant increase in the absolute numbers of CD4^+^ and CD8^+^ T-cells in spinal cord when compared to infected WT mice.

**Fig. 8:**
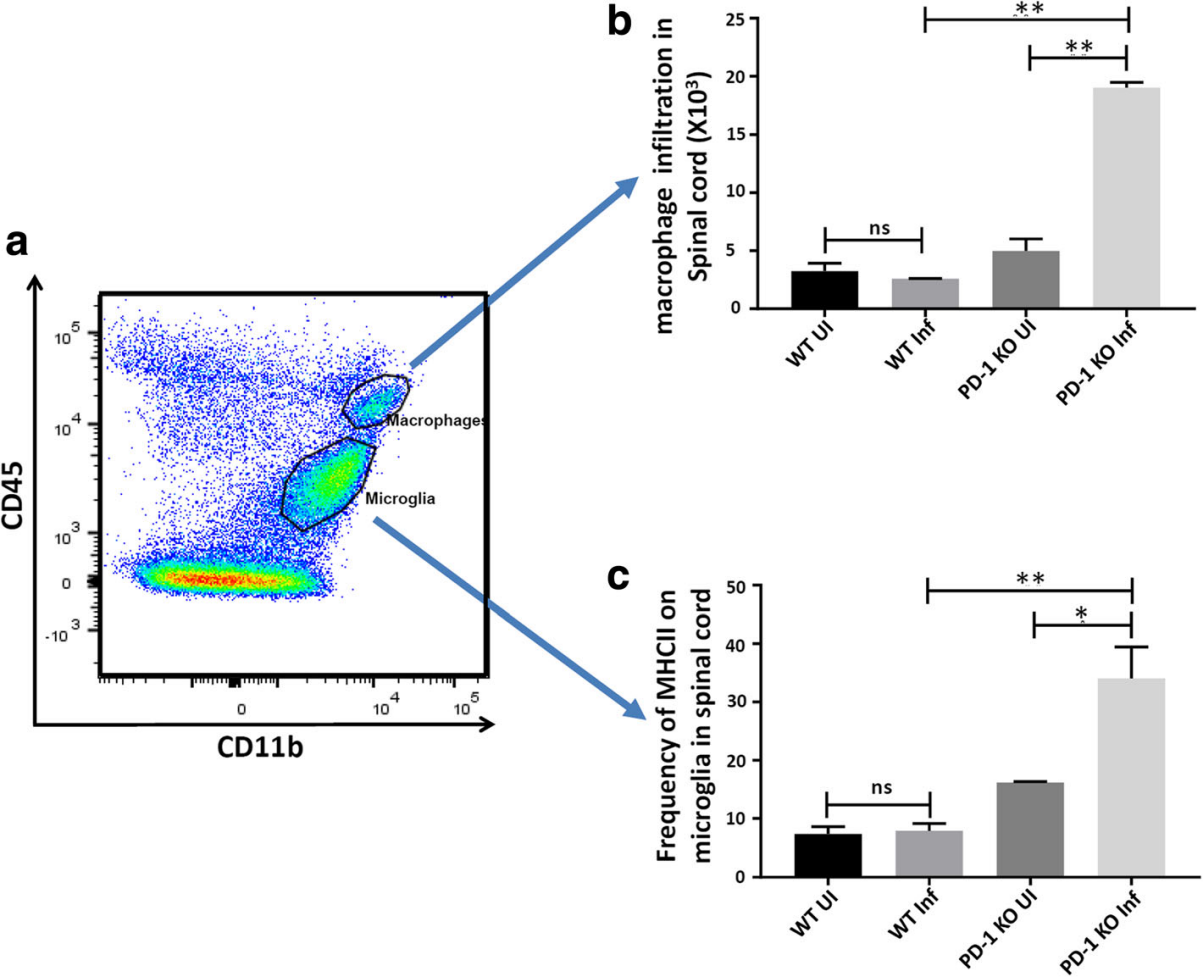
Increased macrophage infiltration and chronic microglial activation in the spinal cord of LP-BM5-infected PD-1 KO animals. **a** Representative dot plot to distinguish infiltrating macrophages from tissue-resident microglia. **b** Absolute numbers of macrophages in the spinal cord of uninfected (UI) and infected (Inf) WT and PD-1 KO animals. Pooled data present absolute numbers (mean ± SD) of infiltrating macrophages within infected and uninfected WT and PD-1 KO animals at 4 weeks p.i. from two independent experiments using three animals per group. **p < 0.01. **c** Pooled data present the frequency (mean ± SD) of microglial cells expressing MHCII in spinal cord tissues of uninfected (UI) and infected (Inf) WT as well as PD-1 KO animals at 4 weeks p.i. *p < 0.05, **p < 0.01

Leukocytes isolated from the spinal cord were subsequently stained with flourochrome-tagged monoclonal antibodies against CD45 and CD11b. Flow cytometric analysis of CD45- versus CD11b-expression can be used to differentiate tissue-infiltrating macrophages from resident microglial cells [28, 43, 44]. Gating on the CD45^int^CD11b^hi^ population identified the microglial cell population, while CD45^hi^CD11b^hi^ cells were identified as infiltrating macrophages (Fig. 8a). The total number of macrophages was enumerated. A significant increase in the absolute number of macrophages infiltrating the spinal cord following LP-BM5 infection in PD-1 KO animals, when compared with WT mice, was observed at 4 weeks p.i. (Fig. 8b). We next assessed activation of resident microglial cells by determining expression levels of MHC class II. We observed a significant upregulation of MHCII expression on microglia (34.0 ± 5.4%) in the spinal cord of LP-BM5-infected PD-1 KO animals as compared with infected WT animals (7.9 ± 1.2%), (Fig. 8c).

### Increased NO-mediated protein damage within the LSC and DRG of LP-BM5-infected PD-1 KO animals

**Fig. 9:**
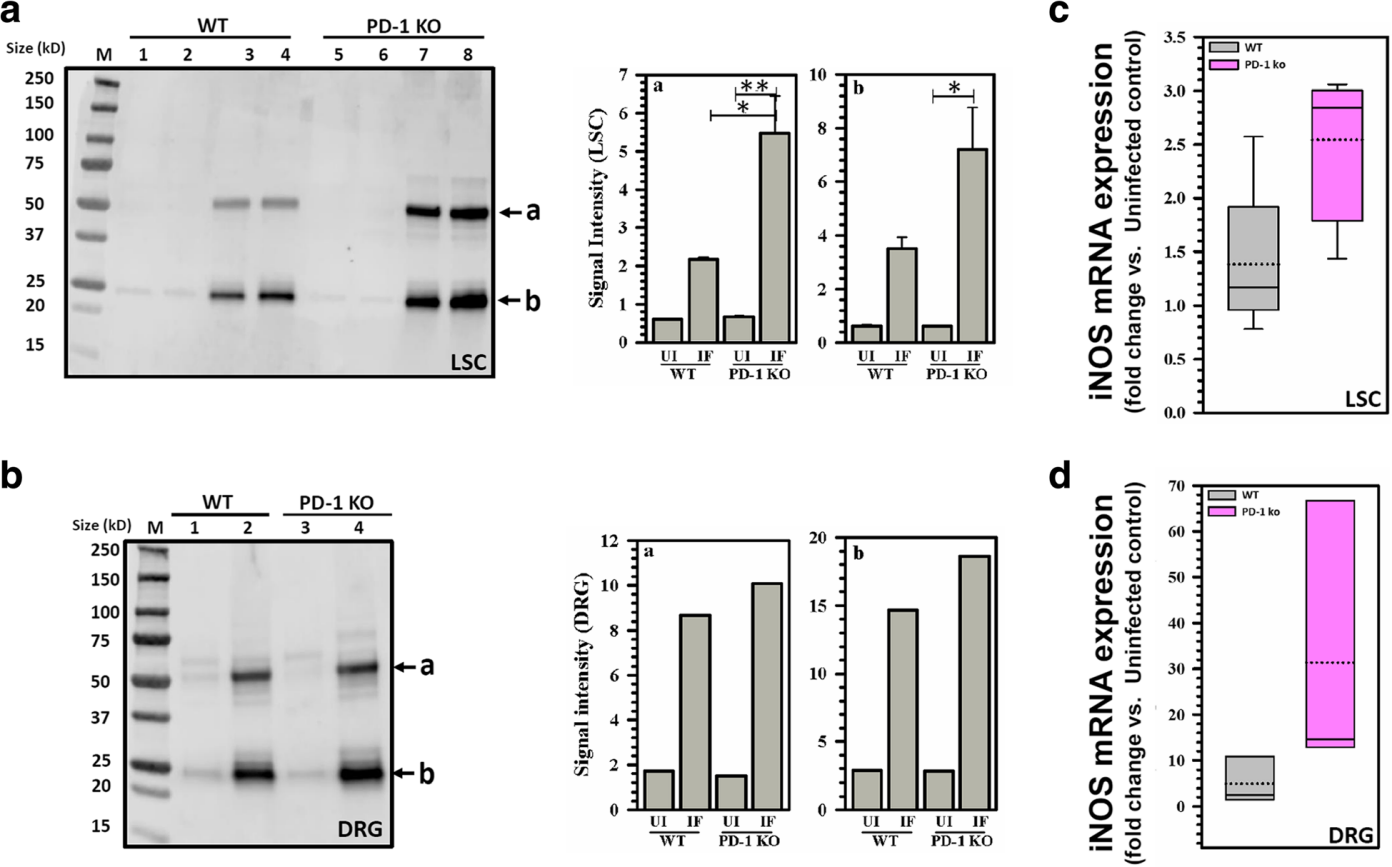
NO-induced damage within the LSC and DRG of LP-BM5 infected PD-1 KO animals. **a** Western blot of LSC tissue lysates from uninfected (UI) and LP-BM5-infected (Inf) WT (UI, lanes 1 and 2; Inf, lanes 3 and 4) and PD-1 KO (UI, lanes 5 and 6; Inf, lanes 7 and 8) animals probed with anti-3-nitrotyrosine antibodies. Each lane displays tissue extract from one individual animal at 10 weeks p.i. The band labeled **a** identifies a high molecular weight (MW) protein band, while the **b** band points out a lower MW protein, both of which are nitrosylated. The signal intensity of both **a** and **b** protein bands observed in the lysates of uninfected and LP-BM5-infected WT and PD-1 KO animals was measured using densitometry and plotted alongside. *p < 0.05, **p < 0.01 (**b**) western blot of lysates obtained from uninfected (UI) and LP-BM5-infected (IF) WT (UI, lane 1 and IF, lane 2) and PD-1 KO (UI, lane 3 and IF, lane 4) animals’ DRG (L3-L5) probed with anti-3-nitrotyrosine antibodies. Each lane displays tissue extract pooled from 3 animals at 10 weeks p.i. The band labeled **a** identifies a high MW protein band, and **b** represents the lower MW protein band that are nitrosylated. The signal intensity of both **a** and **b** protein bands observed was measured and plotted alongside. M = protein molecular weight marker ranging from size 15 to 250 kD. **c** iNOS expression was measured by real-time RT-PCR in the LSC (L3-L5) of LP-BM5-infected WT and PD-1 KO animals at 10 weeks p.i. (*n* = 4–6). **d** iNOS mRNA expression in DRG tissues at 10 weeks p.i. via real-time RT-PCR (*n* = 4–6). Results are presented as box plots of pooled data from individual animals

We next assessed infection-induced NO-mediated protein damage in PD-1 KO animals as compared with WT animals using immunoblotting as described in the previous section. As shown in Fig. 9a, b, we observed nitrosylation of proteins in both WT and PD-1 KO animals infected with LP-BM5 as compared to their age-matched, uninfected littermates in both LSC and DRG. Significantly higher levels of 3-nitrotyrosine were observed in LP-BM5-infected PD-1 KO animals as compared with infected WT animals in LSC (Fig. 9a). However, in the DRG, we observed comparable levels of protein nitrosylation between LP-BM5-infected PD-1 KO and WT animals (Fig. 9b). We observed two major protein bands (labeled a and b) that were nitrosylated in both LP-BM5-infected WT, as well as PD-1 KO animals. Next, we went on to analyze the expression of iNOS in LSC and DRG tissues post-LP-BM5 infection. We observed elevated mRNA levels of iNOS, both in LSC (Fig. 9c) and DRG (Fig. 9d) tissues of LP-BM5-infected PD-1 KO mice, when compared to infected WT animals.

## Discussion

With the advent of effective combination antiretroviral therapy, HIV infection is no more a symbol of imminent death, but rather a chronic disease that is associated with wide-ranging complications; including painful, HIV-associated neural damage. HIV-associated DSP is often underdiagnosed, which can partially be attributed to lack of understanding of its pathophysiology. With an estimated 37 million people living with HIV and 1.8 million new infections in 2016 [45], and with more people gaining access to antiretroviral therapy, the burden of HIV-DSP pain is a problem of enormous global significance. A study by US Department of Veterans Affairs in 2011 showed the prevalence of HIV-sensory neuropathy was 42% among patients at an outpatient clinic in Australia; 92% of patients with sensory neuropathy were on antiretroviral treatment [46]. No routinely available therapy has been shown to be effective for treating HIV-DSP pain. Clearly, there is an urgent need to better understand the pathogenesis of infection-induced HIV-DSP, to develop strategies to prevent this debilitating condition, and to find effective treatments to control its symptoms.

Prior to the induction of cART, DSP in HIV-infected patients was clearly correlated with high plasma viral loads. Hence, in this study, we examined LP-BM5 retroviral load in the LSC and DRG of our infected animals. Viral loads were found to persist within both tissues. However, since the disease has persisted even with early use of cART and well-controlled viral infection, this association has recently become less clear. Paradoxically, patients with HIV-associated neuropathic pain often present enhanced, chronic peripheral immune activation, simultaneous with systemic immunodeficiency. A clear association exists between the diagnosis of low CD4^+^ T-cell count and the development of HIV-associated DSP [47, 48]. In this study, we reported the long-term production of IFN-γ in both LSC and DRG of MAIDS animals, thereby establishing a state of chronic immune activation. Despite these findings, whether development of peripheral neuropathy depends directly on immune activation or viral-induced immunodeficiency has not been investigated in detail. So, the mechanism by which HIV-associated sensory neuropathies develop continues to be the subject of debate. In this study, we investigated the association of immune dysregulation during chronic viral infection with neuropathic pain. Results reported here demonstrate that development of mechanical hind-paw hypersensitivity was closely associated with neuroimmune dysregulation. Recently, it has become apparent that normal immune downregulating mechanisms, such as the PD-1 pathway, limit the magnitude or duration of antiviral T-cell responses. Therefore, strategies to inhibit such negative regulation, and thereby improve protective T-cell immunity, are attractive. However, overzealous immune responses generated by blocking these negative checkpoints may also be responsible for neuroimmune pathology, including but not limited to increased hypersensitivity as demonstrated in this study.

Data presented here show that WT as well PD-1 KO mice infected with LP-BM5 displayed behavioral signs (i.e., mechanical allodynia) of peripheral neuropathy post-infection. We focused on hind-paw hypersensitivity because development of HIV-associated peripheral neuropathy often starts in the lower limbs. Patients with HIV-sensory neuropathies typically present classical distal bilateral sensory symptoms of an axonal, length-dependent polyneuropathy, in a “stocking and glove” distribution with the feet being first affected. Most often, distal regions of the nerve fibers are first affected, with changes eventually progressing proximally [18]. We cannot negate behavioral or pathological changes in the fore paws, but they were not investigated.

Further experiments went on to determine if the increased mechanical hypersensitivity observed in MAIDS animals was associated with increased cellular infiltration and microglial activation. We have previously found that exacerbated neuroinflammation following immune reconstitution was associated with lethality in LP-BM5-infected animals [26]. Neuroinflammation has been implicated in several non-infectious, neuropathic pain models including traumatic nerve injury and diabetic neuropathy [49]. While, T-cell effector responses play a crucial role in protecting against viral infections [50], they are also involved in pain pathology [51]. It has previously been shown that T-cells are essential for the amelioration of paclitaxel-induced neuropathic pain [52]. In addition, animals without T lymphocytes (i.e., nude mice and rats) are known to exhibit significantly reduced mechanical hypersensitivity in nerve injury models. It is also well-established that CD4^+^ T-cells infiltrate the spinal cord and contribute to development of neuropathic pain [53]. In accordance with the cited literature, we showed increased infiltration of T-lymphocytes in the spinal cord and DRG of LP-BM5-infected animals experiencing mechanical hypersensitivity. Similar experiments were carried out that demonstrated the increased mechanical hypersensitivity observed in PD-1 KO animals at 4 weeks post-LP-BM5 infection was associated with increased immune cell infiltration.

Numerous studies have demonstrated the role of activated microglia in development of HIV-associated neurological disorders [54–59]. Correspondingly, studies have also shown that spinal cord glial activation, and their subsequent production of proinflammatory cytokines, can contribute to development of sensory hypersensitivity (a behavioral sign of peripheral neuropathy) [60, 61]. Glial cells have been reported to produce pro-inflammatory molecules capable of contributing to LP-BM5-induced neuronal damage [20, 62]. Moreover, it has been reported that glial cells play a major role in the modulations of pain mechanisms in the spinal cord where there is communication between neurons and microglia. During nerve injury, P_2_X_4_ receptors on microglia are activated by ATP and release brain-derived neurotrophic factor (BDNF) which, through the activation of neuronal TrkB receptors, alters neuronal excitability. This results in the development of behavioral ipsilateral allodynia [63]. MHC class II expression on microglia is considered a surrogate marker for microglial activation [25]. We went on to analyze microglial cells for activation by detecting MHC class II expression. Our study demonstrated elevated levels of MHCII mRNA (~ 3-fold) in the spinal cord of LP-BM5-infected animals by RT-PCR as well as the frequency of microglial cells expressing MHCII was higher (~ 20-fold) as measured by flow cytometry. The stability of mRNA is different than that of proteins, MHCII mRNA was also measured in the whole spinal cord while in the flow cytometry experiments, only microglial cells were analyzed for MHCII expression. As expected, the level of microglial activation was found to be higher in PD-1 KO animals.

Increasing attention is being focused on non-neuronal mechanisms involving immune cells that may amplify or resolve chronic pain [64]. Cells and cytokines conventionally believed to act as coordinators of inflammatory responses are becoming well-accepted as modulators of pain signaling [65, 66]. Viral infections induce neuroinflammation through activation of immune cells, such as macrophages and microglia followed by secretion of neuro-modulatory substances that enhance neuronal excitability and generate pain hypersensitivity [60]. While HIV itself does not replicate in neurons, neuropathological studies have demonstrated the presence of proviral DNA, mRNA, and p24 antigen within macrophages in peripheral nerves [67–69], and in DRG of HIV-infected patients [70, 71]. In addition to T-cells, macrophage infiltration into the peripheral nerves and DRG has been reported in HIV-DSP, as well as other sensory neuropathies [72, 73]. Therefore, we investigated macrophage infiltration into the spinal cord of our LP-BM5-infected mice [65]. The results obtained clearly show significant increase in the macrophage infiltration in LP-BM5-infected PD-1 KO animals exhibiting symptoms of DSP at 4 weeks p.i.

Oxidative damage to the CNS is a well-established consequence of viral brain infection. It has been previously shown that activated microglia are the major source of inducible NO synthase (iNOS) and mediate neuronal injury [74, 75]. In this study, we measured expression of iNOS mRNA in the LSC and DRG tissues of infected animals and as expected we observed higher expression of iNOS mRNA in infected PD-1 KO animals, which supports the neuronal damage to these animals. It has also been shown that brain sections obtained from patients with AIDS dementia show intense immunostaining for nitrotyrosine, indicating that reaction between NO and superoxide has led to peroxynitirite (ONOO-) formation [76]. Moreover, nitrotyrosine has been demonstrated as a new therapeutic biomarker for peripheral diabetic neuropathy, and there are increasing evidence for the role of nitrosative stress in the development of early neuropathy [77–80]. We next assessed NO-induced damage by investigating the presence of nitrotyrosine in the LSC and DRG of animals infected with LP-BM5 exhibiting neuropathic pain by using anti-nitrotyrosine antibody. This antibody stains all proteins with tyrosine moieties that have been nitrosylated. The extent of protein nitrotyrosine formation provides an index of the production of reactive nitrogen species and potential cell damage. In our study, we observed the presence of two major proteins that were nitrosylated post-LP-BM5 infection. These proteins need to be further characterized to obtain insight on their role in neuronal damage post-LP-BM5 infection. These experiments revealed elevated levels of 3-nitrotyrosine within LSC and DRG of infected animals; indicative of peroxynitrite, as well as other nitrogen-centered oxidant-induced protein damage. To gain insight into infection-induced neuronal damage, we stained DRG sections for binding of IB4 or NF200 [8], along with 3-nitrotyrosine [81]. These double-labelling studies demonstrated that 3-nitrotyrosine was present in both small IB4^+^ (non-peptidergic, unmyelinated afferent cell bodies) and NF200^+^ (large myelinated afferent cell bodies) neurons. These data indicate that LP-BM5 infection caused damage to both small and large neurons in our animal model. Further, infected PD-1 KO animals displayed greater expression of 3-nitrotyrosine as compared to WT animals early during infection, which correlated with their greater hind-paw mechanical hypersensitivity.

Currently, there are no FDA-approved pharmacologic agents available which are specifically designed for treatment of chronic HIV-associated neuropathy. The analgesics currently used target neurons (i.e., opioids), but these drugs are only modestly effective for chronic pain and have significant CNS side effects [82]. Our study supports the approach of using inhibitors of microglial activation to limit immune activation-induced neuropathic damage and, correspondingly, ameliorate peripheral neuropathy [83].

Our findings demonstrated that during retroviral infection, circulating CD4^+^ and CD8^+^ T-cells, as well as macrophages, cross the blood-nerve barrier and enter the spinal cord to perform surveillance functions. This cellular infiltration, along with reactive gliosis, is associated with increased neuropathic pain resulting in neuronal damage. However, these are certainly not the sole factors and the mechanistic linkage between cellular infiltration and pain remains to be determined. In subsequent studies we will investigate mechanisms driving neuronal damage and the development of neuropathic pain.

## Conclusions

Although, it has previously been reported that LP-BM5 infection results in enhanced mechanical hypersensitivity to pain [18], in this study, we demonstrated the linkage between neuropathic pain and nitrosative damage to the neurons in LSC and DRG. Furthermore, knocking out the PD-1: PD-L1 negative checkpoint pathway was found to aggravate pain sensitivity. The observed mechanical hypersensitivity in LP-BM5-infected animals was found to be associated with increased infiltration of CD4^+^ and CD8^+^ T-cells, increased macrophage infiltration, and reactive gliosis in the spinal cord. These findings will help further elucidate mechanisms underlying HIV DSP and may be used to test candidate therapeutics.

## Abbreviations

cART: Combination antiretroviral therapy
CGRP: Calcitonin gene-related peptide
DRG: Dorsal root ganglion
DSP: Distal symmetrical polyneuropathy
HIV: Human Immunodeficiency virus
i.p.: Intraperitonial
Iba: Ionized calcium binding adaptor molecule
IENF: Intraepidermal nerve fiber
IHC: Immunohistochemistry
LSC: Lumbar spinal cord
MAIDS: Murine-acquired immunodeficiency syndrome
p.i.: Post infection
PD: Programmed death
PGP 9.5: Protein Gene Product 9.5
WT: Wild-type
